# Quantitative whole-body dynamic planar scintigraphy in mice with ^99m^Tc and ^161^Tb

**DOI:** 10.1186/s40658-025-00775-y

**Published:** 2025-07-01

**Authors:** John D. Wright, Isaline Renard, Isis A. Middleton, Juozas Domarkas, Émer M. Foyle, Paul J. Lusby, Stephen J. Archibald

**Affiliations:** 1 School of Biomedical Engineering and Imaging Sciences, King’s College London, St Thomas’ Hospital, 4th Floor Lambeth Wing, London, SE1 7EH UK; 2 Centre for Biomedicine and PET Research Centre, Hull York Medical School, University of Hull, Hull, HU6 7RX UK; 3 EaStCHEM School of Chemistry, University of Edinburgh, Joseph Black Building, David Brewster Road, Edinburgh, EH9 3FJ Scotland, UK

**Keywords:** Quantification, Dynamic, Planar scintigraphy, SPECT, Gamma camera, Preclinical, Whole-Body, Supramolecular cages

## Abstract

**Background:**

Planar scintigraphy remains commonplace in clinical practice and has been used for quantification and dosimetry estimation over an expanding range of gamma-emitting radionuclides in recent years. Applications of planar scintigraphy, in combination with SPECT/CT imaging, can add value to radiopharmaceutical development in preclinical models and in translation to human use. The aim of this study was to demonstrate whole-body quantitative accuracy in mice using pinhole collimated planar scintigraphy on a preclinical SPECT/CT system, following corrections to sensitivity variations across the field of view.

**Results:**

Planar projections were acquired using short imaging time frames, thus allowing for dynamic biodistribution data to be collected and compared to the known injected activity and whole-body SPECT data. Encapsulation of [^99m^Tc]TcO_4_^–^ in a supramolecular cage was used to demonstrate the visual and quantitative changes in biodistribution over time, as compared to [^99m^Tc]TcO_4_^–^ alone. For these radiopharmaceuticals, whole-body quantification was 98.7 ± 7.3% of the decay-corrected true injected activity, as opposed to 74.8 ± 7.5% when calculated without a sensitivity correction. Similarly, the final planar scintigraphy frame acquired at 1-hour post-injection quantitatively agreed with activity values returned from the whole-body SPECT: 99.5 ± 10.6% (final frame, planar) vs. 99.1 ± 5.5% (SPECT). Regions of interest (ROIs) over selected organs between planar scintigraphy and SPECT were also in good agreement. Quantitative accuracy of planar scintigraphy was further validated in a preclinical tumour model of prostate cancer using [^161^Tb]Tb-PSMA-617. In this case, the whole-body planar value was 94.6 ± 3.6% of the recorded injected activity and, consistent with ^99m^Tc findings, was underestimated without sensitivity correction (76.6 ± 3.1%). Tumour uptake values were equivalent between corrected planar scintigraphy (5.2%IA) and SPECT (5.3%IA) at 1-hour post-injection.

**Conclusions:**

Using a common radionuclide and one of emerging radiotherapeutic interest, whole-body injected activity and organ-specific ROI values obtained by planar scintigraphy strongly correlated to the true injected activity and values obtained by SPECT following sensitivity-based corrections. The addition of quantitative dynamic planar scintigraphy into the preclinical workflow followed by SPECT imaging adds value to pharmacokinetic and dosimetry assessments of novel gamma-emitting radiopharmaceuticals in imaging and therapeutic applications.

## Background

Planar scintigraphy has been used to visualise the accumulation of gamma-emitting radionuclides throughout the body since the inception of the rectilinear and Anger gamma cameras [1]. Since then, advancements in gamma camera technology have enabled whole-body and dynamic imaging protocols in humans, whilst improving quantitative accuracy [2–4]. Despite quantitative scintigraphy data gaining attention in the remit of molecular radiotherapeutic dosimetry, clinical planar imaging remains a popular and reliable tool compared to single-photon emission computed tomography (SPECT), with quantitative methods being developed for planar and SPECT alike [4, 5]. Additionally, it has been suggested that dosimetry estimates from ^177^Lu radioligand therapy returned from planar projections and reviewed in combination with SPECT offer improved accuracy compared to each technique used in isolation [6].

Small animal models are a key preclinical validation tool in the translation of novel radiopharmaceuticals to clinic, including radiotherapeutics containing isotopes such as ^177^Lu and ^161^Tb. However, efforts to incorporate quantitative dynamic planar scintigraphy in combination with SPECT imaging have not been fully realised at the preclinical stage. Preclinical in vivo biodistribution of such radiopharmaceuticals is often achieved by lengthy SPECT methods alone rather than the inclusion of planar scintigraphy, thus sacrificing the potential for dynamic data in favour of 3D information for improved quantitative accuracy.

Applications of dynamic scintigraphy in preclinical models tend to focus on individual organs, e.g. brain, as dedicated collimators bring limitations in field of view (FOV) [7]. This often precludes the ability to acquire whole-body dynamic data, which may be overcome using single pinhole collimators with mice. Single pinhole collimated images suffer from sensitivity loss away from the centre FOV, which complicates a wider quantitative assessment of the whole mouse body without the aid of reconstruction or post-processing corrections specific to planar scintigraphy data. By applying sensitivity-based corrections across the FOV, the count rate can be normalised and quantified, offering whole-body quantification in mice using short imaging time frames. Incorporating quantitative, whole-body dynamic planar imaging into the preclinical SPECT imaging workflow could offer additional value to the radiopharmaceutical development pipeline by providing quantitative data at earlier time points.

The aim of this paper is to demonstrate whole-body and regional quantitative accuracy in mice using planar scintigraphy methods developed on a preclinical SPECT scanner. The proposed methods are validated in vivo using radiopharmaceuticals containing ^99m^Tc, the most commonly used radionuclide in nuclear medicine worldwide, and ^161^Tb, a radionuclide of emerging therapeutic interest, with emission characteristics suitable for scintigraphy.

## Methods

### Radionuclide measurements

A Capintec CRC-15R was used for all radionuclide measurements, which were carried out by suspending the source of interest from the central ring of the well dipper to maintain a consistent spatial orientation and geometry [8]. Where possible, all measurements were made using plastic containers and appropriate calibration factors were confirmed for each radionuclide against the decay-corrected values referenced from the radionuclide source. The ^161^Tb radionuclide and half-life were manually entered into the Capintec database. From the decay corrected calibration reference for ^161^Tb, the calibration factor was experimentally determined using dilutions of known radioactivity ranging from 1 GBq to 2.5 MBq. The precise half-lives used for ^161^Tb and ^99m^Tc were adjusted from values reported in seconds for all radionuclide calibration, instrumentation and data analysis applications.

### Radiochemistry

[^99m^Tc]TcO_4_^−^ was eluted in saline from a ^99^Mo/^99m^Tc generator (Curium). Where high activity/volume ratios were required, the [^99m^Tc]TcO_4_^−^ elution was concentrated following the protocol reported by Blower [9]. A Co_4_^III^L_6_ tetrahedral supramolecular cage (BAB cage), where L is a bis-amino-bipyridine (BAB) ligand, was used to encapsulate [^99m^Tc]TcO_4_^−^ [10]. [^99m^Tc]TcO_4_^−^BAB cage was prepared by addition of [^99m^Tc]TcO_4_^−^ to a solution of BAB cage (40 µM) in water. Encapsulation of [^99m^Tc]TcO_4_^−^ was confirmed by radio-instant thin layer chromatography (iTLC) using silica gel iTLC plates and eluted in water, where free [^99m^Tc]TcO_4_^−^ migrates with the solvent front and [^99m^Tc]TcO_4_^−^BAB cage remains on the baseline.

The commercial stock of [^161^Tb]TbCl_3_ (Terthera, Terbium Theranostics) supplied in 0.05 N HCl was diluted to 0.02 N HCl prior to radiolabelling experiments. [^161^Tb]Tb-PSMA-617 was prepared by addition of 2 µg of precursor to a solution of [^161^Tb]TbCl_3_ (100 µL, 0.02 N HCl). The pH was adjusted to 6 by addition of 0.1 M NH_4_OAc (50 µL) and the reaction was carried out at 80 °C for 30 min with gentle shaking. The reaction mixture was cannulated through a ^t^C18 cartridge, pre-conditioned with 1 mL EtOH, followed by 5 mL saline. The cartridge was washed with 5 mL saline, then eluted with 50 µL EtOH, followed by 50 µL EtOH + 450 µL saline. The bulk of the radioactivity was found in the second fraction. To combat radiolysis, sodium ascorbate (5 mg/mL, final concentration) was added to the formulated radiotracer. Incorporation was determined by radio-iTLC, using silica gel iTLC plates, eluted with 0.1 M sodium acetate/25 mM EDTA (pH = 5.5).

### Scanner

Image data were acquired using a small animal, dual detector nanoSPECT/CT system (Mediso, Hungary). Each detector consists of a 262 × 255 × 6.35 mm thallium-doped sodium-iodide (NaI(Tl)) crystal, connected with a hexagonally-arranged photomultiplier tube array. A 20% energy window over each photopeak of interest was used for data collection, with a single photopeak centred at 140 keV for ^99m^Tc (*t*_1/2_ = 6 h), and two photopeaks centred at 48 and 75 keV for ^161^Tb (*t*_1/2_ = 7 d).

### Planar scintigraphy

Planar scintigraphy images were acquired with one detector using a single pinhole collimator positioned stationary above the imaging cell. For both ^99m^Tc and ^161^Tb, sensitivity measurements were made by collecting the count rate from a concentrated point source at 5 mm intervals across the axial FOV, resulting in a profile encompassing a quantifiable FOV of approximately 7 cm. 600- and 60-second acquisitions were performed to confirm consistent count rates over longer and shorter imaging timeframes. Sensitivity profiles were derived from the count rates using Eq. 1. Correction factors were applied relative to the sensitivity value measured in the centre FOV to achieve count rate uniformity across the FOV.

$$\:\text{S}\text{e}\text{n}\text{s}\text{i}\text{t}\text{i}\text{v}\text{i}\text{t}\text{y}\:\left(\frac{\text{c}\text{p}\text{s}}{\text{M}\text{B}\text{q}}\right)=\frac{\text{m}\text{e}\text{a}\text{s}\text{u}\text{r}\text{e}\text{d}\:\text{c}\text{p}\text{s}\:-\:\text{b}\text{a}\text{c}\text{k}\text{g}\text{r}\text{o}\text{u}\text{n}\text{d}\:\text{c}\text{p}\text{s}}{\text{a}\text{c}\text{t}\text{i}\text{v}\text{i}\text{t}\text{y}\:\left(\text{M}\text{B}\text{q}\right)}$$

**Eq. 1.** Sensitivity calculation. cps = counts per second.

Quantification factors were determined from the linear relationship between count rate and decay-corrected activity. For each radionuclide, a 5 mL syringe phantom containing 1.5 mL of radioactivity was placed in a water jacket, mimicking the attenuation profile of mice, and repeatedly imaged at the centre FOV over 6 half-lives for ^99m^Tc and 3 half-lives for ^161^Tb. Quantitative planar acquisitions were made at 600- and 60-second frames to confirm consistent sensitivity and quantitative accuracy over longer and shorter frame times. The lower limit of quantification was defined as the activity level at which the background count rate begun to dominate over the true count rate, determined from the point at which non-background-corrected sensitivity values deviate from a constant during radioactive decay.

Using ^99m^Tc, the application of sensitivity correction and quantification factors were tested in phantoms comprising of 5 equally distanced vials across the FOV, each containing 500 µL of equal radioactivity (11.7 ± 1.0 MBq). Planar scintigraphy images were acquired over 10 min. Count rates were taken from regions of interest (ROIs) drawn over each vial, and sensitivity correction factors were applied to each ROI based on their respective axial position, followed by quantification.

### SPECT

Energy, uniformity and quantification calibrations were performed for all radionuclides following manufacturer guidelines. Whole-body SPECT imaging in mice was performed using 2 detectors equipped with multi-pinhole collimators over *ca*. 30 min (30 s per frame) and accompanied by computed tomography (CT) for anatomical co-registration. SPECT images were reconstructed using an ordered subset expectation maximisation (OSEM) iterative reconstruction algorithm (HiSPECT, Scivis GmbH, Germany). The versions of software used did not allow for CT derived attenuation correction to be applied to SPECT reconstructions. Similar to the method described for planar scintigraphy quantification, syringes containing known activities of each radionuclide were placed in a water jacket to mimic the attenuation profile of a mouse to generate quantification factors which were then input directly into the SPECT reconstruction software.

### Animal models

Female SWISS and male BALB/c nude mice (Janvier Laboratories) were allowed to acclimatise for a minimum of 1 week before study commencement. Food and water were provided *ad libitum*. PSMA-expressing LNCaP cells (7.5 × 10^6^) in Matrigel (Corning)/PBS (50:50) were injected subcutaneously (100 µL) over the right shoulder of male BALB/c nude mice. Once palpable, tumours were measured twice weekly by calliper. Radiotracer administration and imaging studies were performed once tumour burden reached *ca*. 400 mm^3^.

### In vivo imaging

Anaesthesia was induced and maintained using 5 and 2% isoflurane, respectively, in a 1 L/minute oxygen flow. Mice were cannulated (tail vein) and transferred to the imaging cell, where temperature and respiration were monitored throughout scanning. Animals were centred in the FOV under a single pinhole collimator with aid of an x-ray scout scan. Once positioned, 60-second planar scintigraphy frames were repeatedly acquired over 1 h. The first acquisition was synchronised with radiotracer administration. Animals were injected (200 µL) with either [^99m^Tc]TcO_4_^−^ (18.5 ± 2.7 MBq, *n* = 11), [^99m^Tc]TcO_4_^−^BAB cage (18.0 ± 3.6 MBq, *n* = 11), or [^161^Tb]Tb-PSMA-617 (22.7 MBq, *n* = 1). For animals that underwent SPECT/CT immediately after the dynamic planar scintigraphy (*n* = 3 [^99m^Tc]TcO_4_^−^, *n* = 3 [^99m^Tc]TcO_4_^−^BAB cage and *n* = 1 [^161^Tb]Tb-PSMA-617), the single pinhole collimator was exchanged for a multi-pinhole collimator and a 30 min whole-body SPECT/CT scan was acquired. Syringes and catheters containing radiotracers were measured before and after injection to calculate the injected activity.

### Image analysis

All image data were saved in DICOM format and analysed using VivoQuant (InVicro, USA). Sensitivity profiles and quantification measurements were made from count-rate data extracted from the whole FOV. ROIs with a width of 5 mm, corresponding to the sensitivity profile intervals, were drawn along the axial FOV of the 5-vial phantom and in vivo planar scintigraphy datasets and sensitivity correction factors were applied to count rates extracted from each ROI. Quantification of the summed count rates returned sensitivity-corrected whole FOV activity (in MBq), which correlates to the whole-body injected activity as shown in Eq. 2.

$$\:\text{I}\text{n}\text{j}\text{e}\text{c}\text{t}\text{e}\text{d}\:\text{a}\text{c}\text{t}\text{i}\text{v}\text{i}\text{t}\text{y}=\:\frac{\sum\:_{\text{i}=1}^{\text{n}}\left(\frac{{\text{c}}_{\text{i}}\:\times\:{\text{S}\text{F}}_{\text{i}}}{{\Delta\:}\text{t}}\right)-\text{B}\text{G}}{\text{Q}}$$

**Eq. 2.** Equation used to determine whole-body radioactivity (in MBq) from planar acquisition, where n is the total number of sensitivity-based ROIs across the FOV; c_i_ is the raw counts obtained for each ROI; SF_i_ is the sensitivity correction factor for the specific ROI; Δt is the frame time in seconds; BG is the background counts in the whole FOV and Q is the quantification factor for a given radionuclide.

Whole-body injected values derived from planar scintigraphy were compared to the calculated injected activities and those derived from whole-body SPECT. In a method similar to that previously described for clinical applications of planar scintigraphy quantification, comparisons were reported as the percentage of the recorded injected activities ± the standard deviation, where 100% represents perfect quantitative accuracy, values less than 100% are an underestimation, and values greater than 100% are an overestimation [4].

ROIs were hand drawn over selected organs and sensitivity and quantification factors were applied to the extracted count rates. Organ activity values were normalised to the recorded injected activity and reported as the percent injected activity (%IA). Similarly, volumes of interest (VOIs) were hand drawn over selected organs of reconstructed SPECT images, guided by the anatomical boundaries as visualised by co-registered CT. VOIs were reported as %IA for direct comparison to planar scintigraphy data. SPECT images were scaled and reported using standard uptake values (SUV).

## Results

### Planar scintigraphy phantoms

**Fig. 1:**
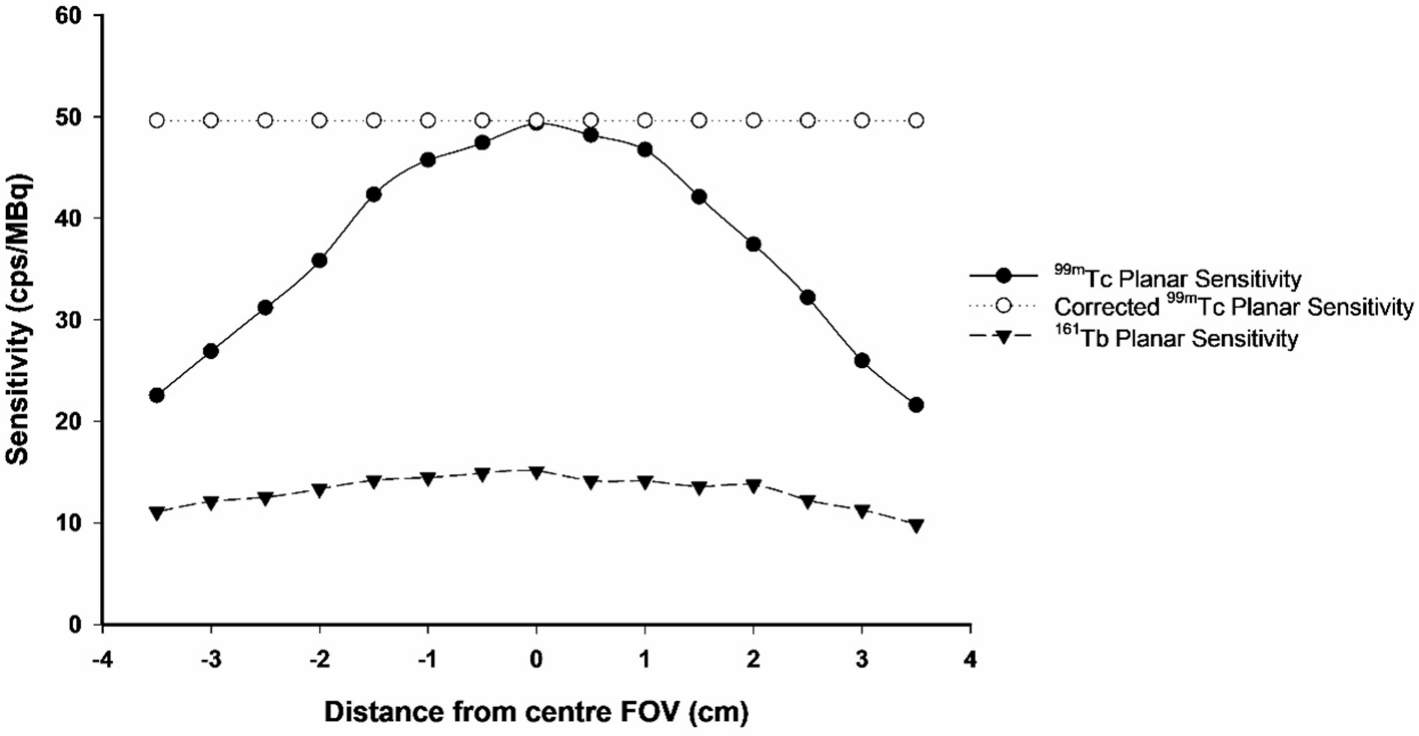
Single pinhole collimator sensitivity profiles for ^99m^Tc and ^161^Tb

The planar sensitivity profile for ^99m^Tc peaked at 50 cps/MBq at the centre FOV and decreased as the source moved toward the edges of the visible frame. A less pronounced yet similar pattern was observed for ^161^Tb, which peaked at 15 cps/MBq (Fig. 1).

**Fig. 2:**
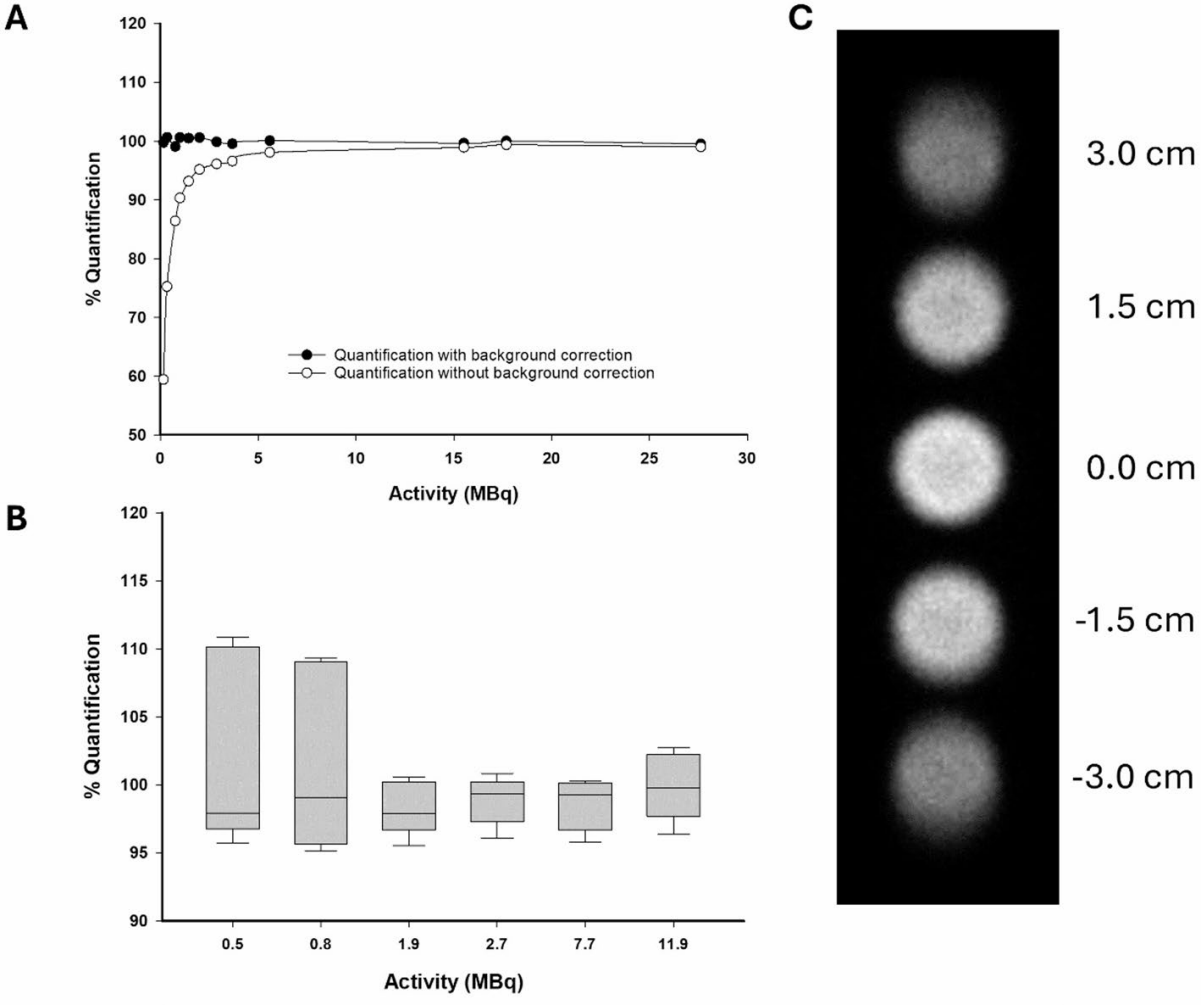
(**A**) Planar quantification accuracy using a syringe phantom, analysed with and without background correction. (**B**) Planar quantification accuracy across the whole FOV using 5-vial phantom, where all vials are equally spaced and contain an equal amount of radioactivity. Data are presented as the median and interquartile ranges. (**C**) Representative planar projection of a 5-vial phantom

Activities returned from planar scintigraphy quantification phantoms were compared to their decay corrected values from the radionuclide measurements, and the accuracy reported as the % difference. No difference in quantitative accuracy was noted when the imaging time varied between 600 and 60 s. For ^99m^Tc, the requirement for background correction increased as the decaying activity approached 5 MBq (Fig. 2A). Sensitivity and quantification factors were tested in a 5-vial phantom design encompassing the whole FOV, with each vial containing an equal amount of radioactivity (11.7 ± 1.0 MBq). From planar projections, loss of signal was visually evident in the peripheral vials and was in line with the sensitivity profile (Fig. 2C). Quantitative accuracy over all 5 vials across the FOV was 98.9 ± 2.0% when the radioactivity in each vial remained higher than 1 MBq (5 MBq whole FOV) following sensitivity correction. In contrast, quantitative accuracy reduced to 67.3 ± 20.5% when sensitivity corrections were not applied. As the radioactivity decayed to values less than 1 MBq, sensitivity corrected quantification begun to trend towards overestimation and with greater error (Fig. 2B).

### In vivo imaging

**Fig. 3:**
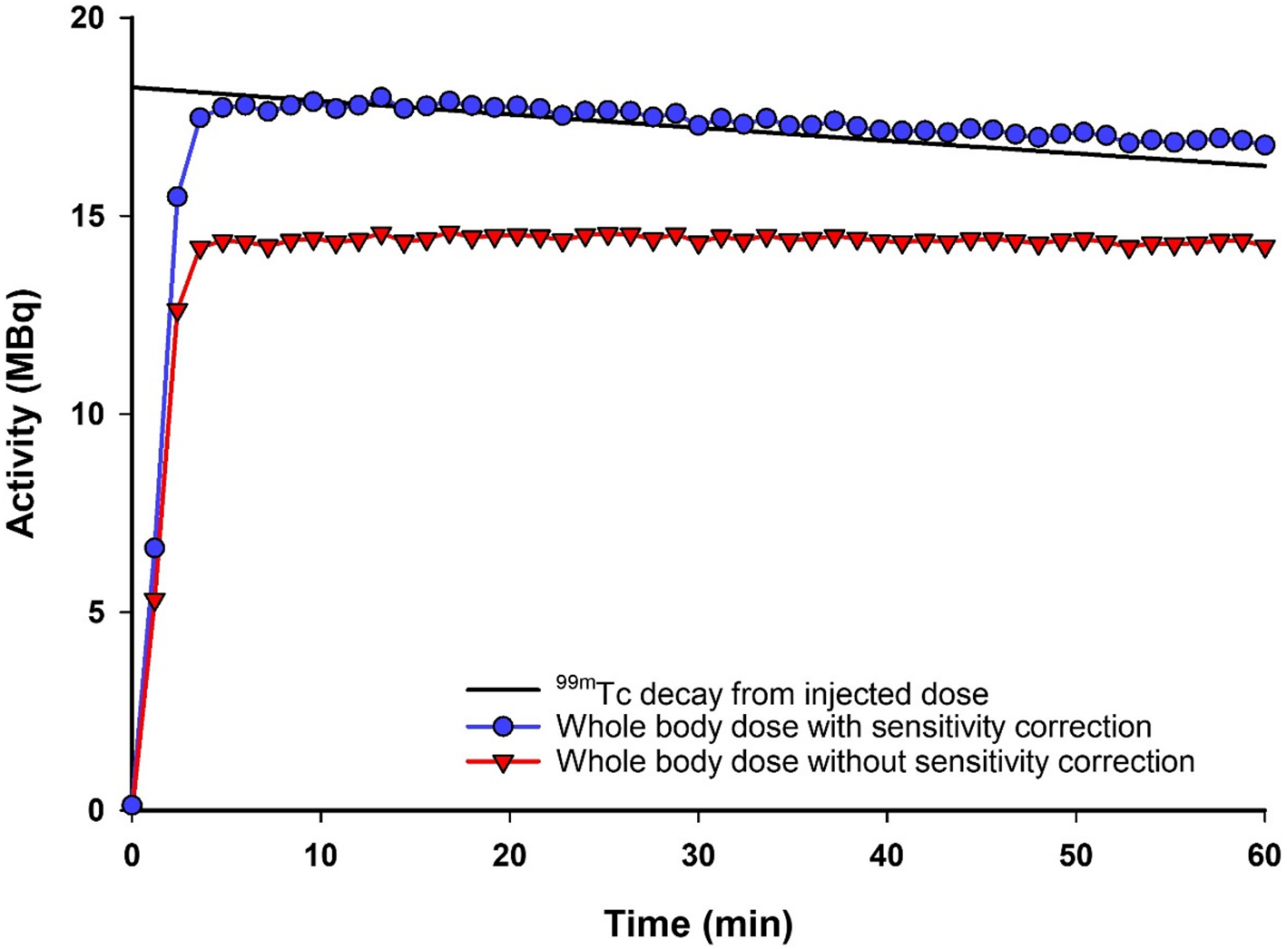
Representative whole-body time activity curve (TAC) of [^99m^Tc]TcO_4_^−^, with and without sensitivity correction, compared to the calculated injected activity

Whole-body quantitative accuracy of 1-minute frames acquired by planar scintigraphy between 5 and 60 min post-injection was 101.1 ± 6.6% and 97.3 ± 8.2% for [^99m^Tc]TcO_4_^−^ (*n* = 11) and [^99m^Tc]TcO_4_^−^BAB cage (*n* = 11), respectively. The same data quantified without sensitivity correction led to a considerable underestimation in whole-body activity, returning 76.6 ± 7.6% and 73.3 ± 6.9% for the same radiopharmaceuticals, respectively (Fig. 3).

**Fig. 4:**
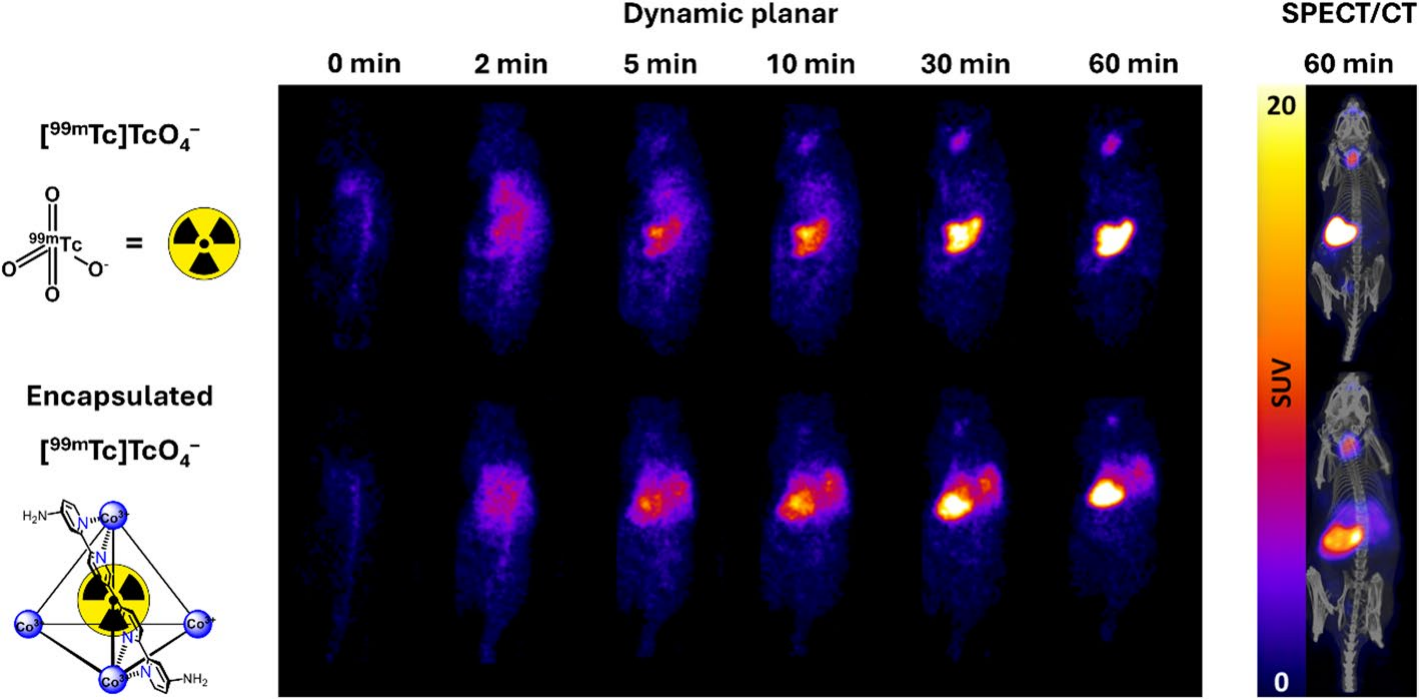
Representative planar scintigraphy and SPECT/CT images of [^99m^Tc]TcO_4_^−^ and [^99m^Tc]TcO_4_^−^BAB cage at selected timepoints

**Fig. 5:**
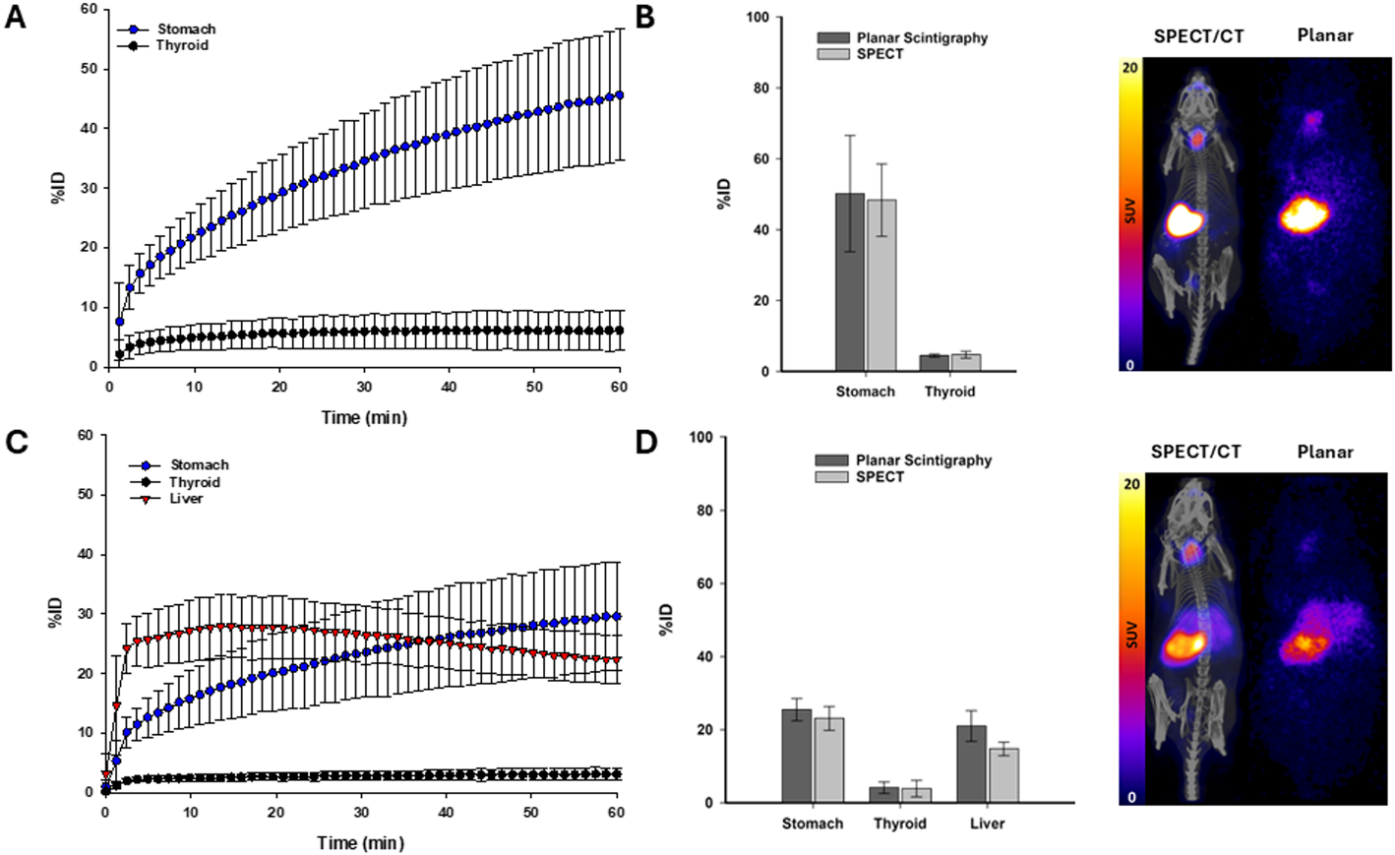
(**A**) Dynamic planar scintigraphy time activity curves of selected organs following administration of [^99m^Tc]TcO_4_^−^. (**B**) Comparison of dynamic planar scintigraphy and SPECT quantification of [^99m^Tc]TcO_4_^−^ accumulation in selected organs at 1 h post-injection and representative scans. (**C**) Dynamic planar scintigraphy time activity curves of selected organs following administration of [^99m^Tc]TcO_4_^−^BAB cage. (**D**) Comparison of dynamic planar scintigraphy and SPECT quantification of [^99m^Tc]TcO_4_^−^BAB cage accumulation in selected organs at 1-hour post-injection and representative scans. Data are shown as mean ± SD

Planar scintigraphy images show uptake in thyroid and stomach in animals injected with [^99m^Tc]TcO_4_^−^ and uptake in thyroid, stomach and liver for animals injected with [^99m^Tc]TcO_4_^−^BAB cage (Fig. 4). [^99m^Tc]TcO_4_^−^ (*n* = 11) scintigraphy at 1 h post-injection showed stomach and thyroid uptake of 45.7 ± 11.1%IA and 6.1 ± 3.4%IA, respectively (Fig. 5A). For the subset of animals (*n* = 3) which underwent dynamic planar scintigraphy followed by SPECT/CT, the stomach and thyroid activities returned by planar scintigraphy at the 1-hour timepoint were 50.2 ± 16.4 and 4.4 ± 0.4%IA and were 48.3 ± 10.2 and 4.7 ± 1.0%IA by SPECT, respectively (Fig. 5B). Decay-corrected whole-body activities represented 101.5 ± 3.8% (SPECT) and 104.9 ± 11.2% (planar scintigraphy) of the recorded injected activities.

[^99m^Tc]TcO_4_^−^BAB cage (*n* = 11) scintigraphy at 1-hour post-injection returned stomach, thyroid and liver values of 29.6 ± 9.2%IA, 3.0 ± 1.1%IA and 22.3 ± 4.0%IA, respectively (Fig. 5C). For the subset of animals (*n* = 3) which underwent dynamic planar scintigraphy followed by SPECT/CT, uptake observed at the 1-hour timepoint of planar scintigraphy was 25.5 ± 3.1%IA (stomach), 4.1 ± 1.6%IA (thyroid) and 21.0 ± 4.2 (liver) %IA. Similarly, uptake observed in SPECT images was 23.1 ± 3.3%IA (stomach), 3.9 ± 2.3%IA (thyroid) and 14.8 ± 1.9 (liver) %IA at 1-hour post-injection (Fig. 5D). Decay-corrected whole-body activities represented 96.7 ± 6.7% (SPECT) and 94.1 ± 8.4% (planar scintigraphy) of the calculated injected activities.

**Fig. 6:**
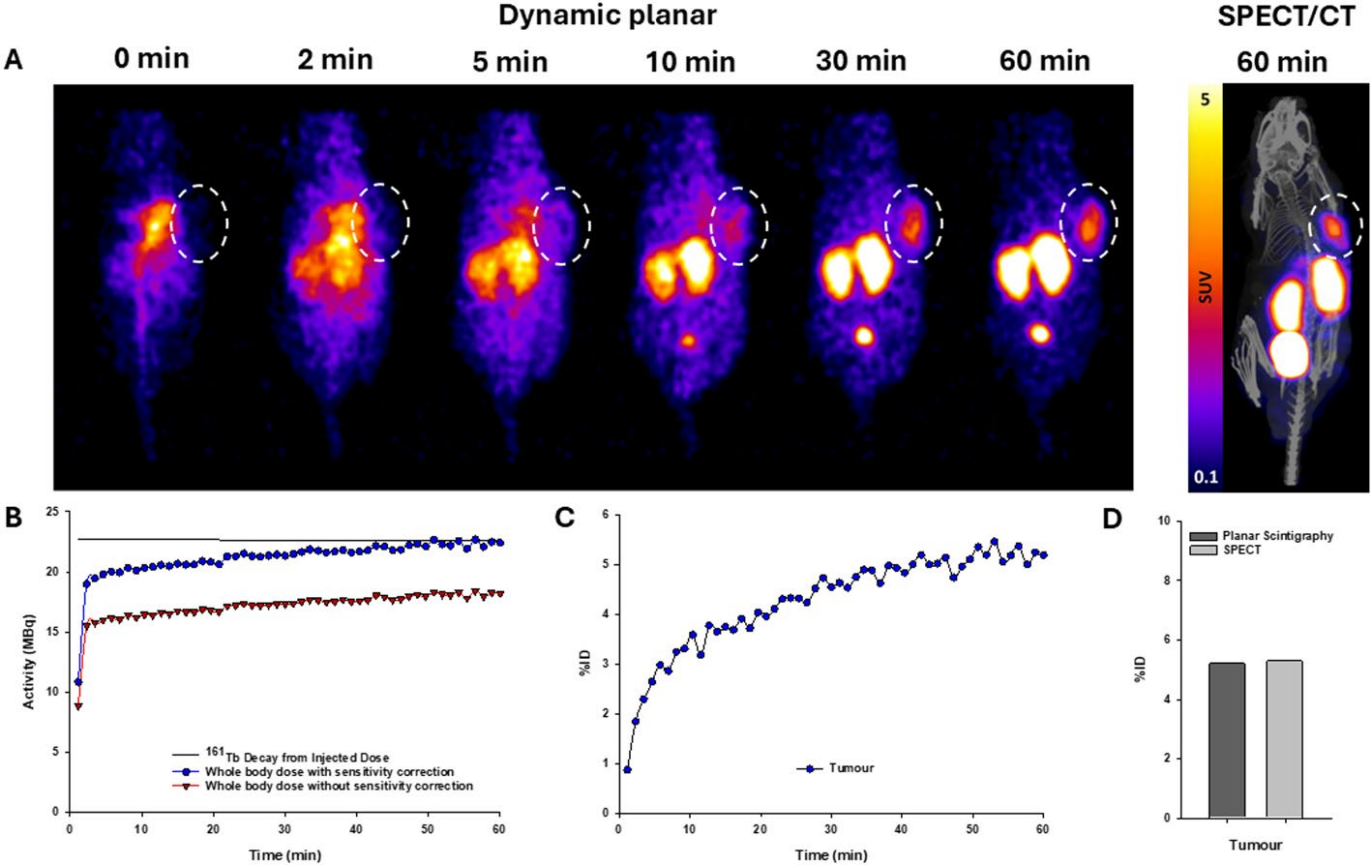
(**A**) Representative planar scintigraphy and SPECT/CT images of [^161^Tb]Tb-PSMA-617 at selected timepoints. The dashed region is positioned over the tumour. (**B**) [^161^Tb]Tb-PSMA-617 whole-body time activity curve, with and without sensitivity correction. (**C**) Dynamic planar scintigraphy time activity curves in LNCaP tumour following administration of [^161^Tb]Tb-PSMA-617. (**D**) Comparison of dynamic planar scintigraphy and SPECT quantification of [^161^Tb]Tb-PSMA-617 accumulation in the tumour at 1-hour post-injection

For [^161^Tb]Tb-PSMA-617 (*n* = 1), increase in tumour uptake was observed over time, with values of 5.2%IA (planar) and 5.3%IA (SPECT) at 1 h post-injection (Fig. 6). The whole-body activity obtained by SPECT was 105.2% of the recorded injected activity. Whole-body quantitative accuracy of 1-minute frames acquired by planar scintigraphy between 5- and 60-minutes post-injection was 94.6 ± 3.6%. The same data quantified without sensitivity correction underestimated whole-body activity with 76.6 ± 3.1% quantitative accuracy.

## Discussion

In recent years, dedicated equipment for whole-body planar scintigraphy in animals has been reported, including the MONICA and GAEDE anger cameras, as well as Bioemtech’s benchtop γ-eye [11–13]. Few systems have demonstrated applications in whole-body dynamic imaging with validated quantification and calibration methods using SPECT/CT as a direct comparator. Bioemtech’s γ-eye offers quantitative, dynamic whole-body planar scintigraphy in mice, comparable to both SPECT imaging and ex vivo biodistribution data, though without the additional workflow of acquiring SPECT/CT images using the same system [11]. The γ-eye offers a reported 92 cps/MBq sensitivity using ^99m^Tc. The improvement in sensitivity over the single pinhole collimated planar scintigraphy method is likely attributed to the multiple parallel hole collimators employed in the γ-eye, as well as the use of denser caesium iodide crystals which provide improved counting efficiencies over sodium iodide.

Whole-body animal imaging, including rat scintigraphy using clinical scanners has been reported [14]. However, the use of clinical equipment for rodent imaging is an impractical solution in routine delivery of preclinical research and comparatively poorer spatial resolution may confound quantification of individual organs. Carlson et al.. compared single timepoint planar images to SPECT, reporting inaccuracies between injected activity determined by radionuclide calibrator and whole-body SPECT. Unfortunately, this work did not take advantage of shorter time-frame dynamic imaging, nor demonstrate quantitative accuracy in planar imaging using established corrections, focusing instead on SPECT quantification [15].

In our preclinical SPECT imaging practice, quantification phantoms are used to generate conversion factors which are measured under controlled radionuclide calibration orientations and geometries, resulting in reproducible and accurate quantification between activity measurements and SPECT readouts for in vivo imaging. Our data demonstrates quantitative accuracy in both SPECT and planar imaging, with validated whole-body quantification referenced to the known injected activity obtained by decay corrected activity measurement. Methods for acquiring dynamic whole-body planar scintigraphy data in mice using SPECT/CT imaging equipment offers a streamlined approach of quantitative biodistribution in radiopharmaceutical development and dosimetry studies which may demand subsequent SPECT timepoints.

Effective implementation of full FOV quantification requires a uniform count rate response regardless of source location. Calibration methods for accomplishing uniformity vary between manufacturers for both preclinical and clinical equipment. Gamma cameras which utilise photomultiplier tubes are often coated with reflective material which guides light towards the tube centre to increase efficiency. However, this generates a distorted *X*,* Y* coordinate grid over the detector face as scintillation light appears to concentrate towards the centre of the photomultiplier tubes. A point source moving across the FOV in the absence of linearity calibration would appear to trace a wave-like line.

The energies for isotopes of interest are also calibrated by tuning the photomultiplier tube voltages. Properly tuned gamma cameras may then be further calibrated for uniformity by addressing features such as pulse-height variations, where inherent differences in photomultiplier tube efficiency are corrected for. From a radioactive uniform flood source, the pulse heights of each photomultiplier tube, which are linked to form the *X*,* Y* coordinate projection data, are stored and compared to subsequent measurements, which are then adjusted for using documented correction factors. Without such corrections in place, the outlines of photomultiplier tubes may appear as shadows in images as the projections fail to correctly weight the position and amplitudes of pulses from scintillation events between the tubes.

Many preclinical gamma cameras use point sources to perform calibrations, mainly due to the reduced, and often static, distance between the source, collimator and detector. Clinical scanners on the other hand typically use flood sources over varying distances from the detector, as the camera faces are often translated closer or farther away from the patient depending on their size, weight and anatomical region of interest. As many calibrations are performed in the absence of collimators, the collimator of choice for subsequent measurements following calibration continues to impact count-rate uniformity, particularly in the instance of single pinhole collimators, as shown in this work. Due to the flexibility in detector distance and the increased impact of attenuation and scatter in clinical imaging as opposed to preclinical scintigraphy, normalisation of count-rate uniformity can be more easily achieved for preclinical models using documented sensitivity-based correction factors, similar to other uniformity calibrations.

In the absence of automated corrections specific to planar projection data, scintigraphy images may appear distorted. However, variations in count rate can be corrected for with factors taken from sensitivity measurements over the FOV, allowing for quantitative analysis. As single pinhole collimated projections from a small animal SPECT/CT system returns a visible FOV large enough to encompass the whole body of mice, whole-body quantification can be achieved in short frames. Regardless of distance from the centre FOV, we report quantitative accuracy following sensitivity correction within 5% of the true activity value in phantoms containing activities greater than 1 MBq. Planar quantification factors were derived from syringe phantoms using a water jacket designed to mimic the attenuation profile of mice, thus inherently applying a crude attenuation correction factor to all quantitative measurements, albeit a single value for all datasets.

The impact of background subtraction on quantification phantoms was used to inform on acceptable injected activity levels, with the goal of minimising reliance on quantitative corrections. Background subtraction consistently accounted for less than 2% of quantitative accuracy for activities greater than 5 MBq, demonstrating that background-related noise levels were sufficiently outweighed by the count rate of the presented radionuclide. In the case of activities below 5 MBq, an increasing reliance on background correction became evident (Fig. 2A).

In vivo datasets collected in this study were all acquired with injected activities greater than 10 MBq, ensuring sufficient count rates to achieve reliable quantification, as well as improved image quality [16]. The compromise of 3D data in favour of 2D dynamic imaging brings challenges in drawing accurate ROIs over organs which overlap in the coronal plane, leading to difficulties in accurately determining organ mass. As such, data is presented in %IA of the whole organ, rather than normalising to mass (%IA/g). SPECT data is also presented in %IA of the whole organ for the purpose of validating the dynamic planar methodology.

Sensitivity correction was necessary to return accurate whole-body injected activities in mice for both ^99m^Tc- and ^161^Tb-labelled radiopharmaceuticals. Due to the first frame acquisition coinciding with injection, the first 5 min were excluded from whole-body analysis to ensure that the full activity was present in the FOV for all datasets analysed. A total of 1042 frames were collected for all ^99m^Tc-based datasets (*n* = 22) acquired between 5 and 60 min. Of these frames, the whole-body activity was 99.3 ± 7.6% of the decay-corrected true injected activity. This value dropped to 75.0 ± 7.4% when no sensitivity correction factors were applied, highlighting the need for sensitivity correction. Of the 6 animals which underwent ^99m^Tc SPECT/CT imaging immediately following 1-hour dynamic planar scintigraphy, there was a remarkable agreement in whole-body accuracy between the two measurements, 99.5 ± 10.6% (final frame, planar) vs. 99.1 ± 5.5% (SPECT).

The use of robust Co_4_^III^L_6_ supramolecular tetrahedral assemblies, such as the BAB cage, for the encapsulation of [^99m^Tc]TcO_4_^−^ was previously reported [10]. This type of metallo-organic cage is stable in vivo and rapidly forms a host-guest complex with radio-anions, [^99m^Tc]TcO_4_^–^ for example, without the need for additional reagents, offering an attractive platform for the future development of kit-based radiopharmaceuticals. We previously reported SPECT/CT in vivo data showing a marked shift in biodistribution between free and encapsulated [^99m^Tc]TcO_4_^−^, resulting in increased liver uptake with [^99m^Tc]TcO_4_^−^BAB cage [10]. However, SPECT alone does not capture the dynamic changes expected with this type of host-guest equilibrium system. Dynamic planar scintigraphy offers an innovative methodology for visualising the in vivo retention of radio-anions in supramolecular cage constructs over time which is essential to the future development and optimisation of this technology.

Compared to free [^99m^Tc]TcO_4_^−^, a marked increase in liver uptake and reduction in stomach and thyroid uptake was observed by planar scintigraphy following administration of [^99m^Tc]TcO_4_^−^BAB cage. Liver activities were seemingly overestimated in planar methods compared to SPECT. Based on planar time-activity curves, uptake in the liver decreases over time. As SPECT acquisitions were carried out over 30 min following the final planar scintigraphy timepoint, it is possible that the reduced liver activity observed by SPECT comes from the continuously decreasing activity in the liver during the acquisition. In this instance, it is unlikely that overlapping organs visualised in 2D planar scintigraphy were responsible for differences in activity estimation, as uptake in the stomach, the major competing organ in proximity of the liver, remained in good agreement between both methods.

Of note, the selected ^99m^Tc-based radiopharmaceuticals display typical biodistributions in the stomach and liver, which lie close to the centre FOV where sensitivity is optimal. Despite this, sensitivity-based corrections were still necessary to prevent around 25% whole-body activity underestimation. It is expected that other radiopharmaceuticals with uptake patterns over more distal organs and tissues (e.g. brain) would suffer to a greater extent, as demonstrated by the 5-vial phantom acquisition used to quantify ROIs across the whole FOV.

The efficacy of ^161^Tb as a radiotherapeutic radionuclide with imaging characteristics has been reported and compared to ^177^Lu. [^177^Lu]Lu-PSMA-617 has been extensively investigated as an effective radiotheranostic and has been successfully translated to human use for the management of advanced prostate cancers [17, 18]. More recently, ^161^Tb has been proposed as an alternative to ^177^Lu, largely owing to the additional Auger electrons emitted alongside beta particles which brings the potential for an improved therapeutic response [19], and a clinical trial using [^161^Tb]Tb-PSMA-I&T is currently on-going (NCT05521412).

Optimised preclinical imaging protocols, such as dynamic planar imaging, using ^161^Tb in the radiopharmaceutical development pathway can support the collection of biodistribution data at earlier timepoints to support the translation of these new radiopharmaceuticals. The low energies and branching ratios of ^161^Tb gamma emissions (48 keV, 17% and 75 keV, 10%) result in poorer imaging sensitivity compared to ^99m^Tc (140 keV, 100%) with values of 15 and 50 cps/MBq for ^161^Tb and ^99m^Tc, respectively, using single pinhole collimators on our system.

Quantitative dynamic planar scintigraphy with ^161^Tb was demonstrated in an LNCaP tumour-bearing mouse using [^161^Tb]Tb-PSMA-617, followed by SPECT imaging (Fig. 6). Similarly to the datasets obtained with ^99m^Tc, the whole-body activity derived from ^161^Tb planar scintigraphy frames acquired between 5- and 60-minutes post-injection (49 frames) was 94.6 ± 3.6% of the decay-corrected true injected activity. This value decreased to 76.6 ± 3.1% without sensitivity correction. Importantly, tumour uptake was in good agreement between scintigraphy (5.2%IA) and SPECT (5.3%IA), highlighting the complimentary applicability of dynamic scintigraphy in combination with SPECT imaging for molecular radiopharmaceutical studies. Clinically, a ± 10% quantitative accuracy has been suggested as an acceptable window for applying dosimetry calculations [4, 20]. It is worth noting that the planar sensitivity profile of ^161^Tb did not vary as dramatically as that of ^99m^Tc when moving away from the centre of the FOV (Fig. 1), yet correction for sensitivity was equally necessary to avoid around 25% underestimation of whole-body activity in vivo.

## Conclusions

In the presented work, the ability to acquire quantitative whole-body dynamic planar scintigraphy images on a small animal SPECT/CT scanner has been demonstrated and incorporated into a SPECT imaging workflow with ^99m^Tc- and ^161^Tb-based radiopharmaceuticals. Sensitivity deteriorates away from the centre of the FOV when acquiring projections using single pinhole collimators and requires sensitivity-based corrections to return quantitative data and avoid underestimations. Whole-body and organ-specific activities can be accurately determined by planar scintigraphy, and adoption of such methods in preclinical SPECT studies adds value in radiopharmaceutical development and dosimetry measurements.

## Data Availability

The datasets used and/or analysed during the current study are available from the corresponding author on reasonable request.

## Abbreviations

%IA: Percent injected activity
%IA/g: Percent injected activity per gram
2D: Two dimensional
3D: Three dimensional
CPS: Counts per second
CT: Computed tomography
FOV: Field of view
iTLC: Instant thin-layer chromatography
OSEM: Ordered subset expectation maximisation
PET: Positron emission tomography
ROI: Region of interest
SPECT: Single-photon emission computed tomography
SUV: Standard uptake value
VOI: Volume of interest
